# Kinetics of early immune reconstitution and its exploratory association with post-transplant outcomes in patients undergoing haploidentical HCT with post-transplant cyclophosphamide-based GvHD prophylaxis

**DOI:** 10.3389/fimmu.2026.1996185

**Published:** 2026-10-26

**Authors:** Nayanthara K. Bijukumar, Aswin Anand Pai, Rachel Mariam Renji, Agila Jayaraman, Raveen Stephen Stallon Illangeswaran, Arun Kumar Arunachalam, Phaneendra D. V., Kavitha M. Lakshmi, Eunice S. Edison, Sushil Selvarajan, Sharon Anbumalar Lionel, Anu Korula, Aby Abraham, Vikram Mathews, Biju George, Sujith Karumathil, Uday Kulkarni, Poonkuzhali Balasubramanian

**Affiliations:** 1 Department of Hematology, Christian Medical College, Vellore, India; 2 Department of Biotechnology-Regional Centre for Biotechnology (RCB), Faridabad, Haryana, India

**Keywords:** graft-versus-host disease, haploidentical HCT, immune reconstitution, post-transplant cyclophosphamide, regulatory T cells, transplant-related mortality

## Abstract

**Introduction:**

Post-transplant cyclophosphamide (PTCy) is widely used for graft-versus-host disease (GvHD) prophylaxis in haploidentical hematopoietic cell transplantation. However, the kinetics of early immune reconstitution in real-world, heterogeneous cohorts, and their relationship with early outcomes, remain incompletely defined.

**Methods:**

In this prospective single-center study, 95 consecutive patients undergoing haploidentical transplantation with PTCy (October 2021 to December 2024) underwent serial multicolor flow cytometry before conditioning, at neutrophil engraftment, and on days +21, +28, +60 and +90. T, B and natural killer (NK) cells were enumerated, along with CD4+ and CD8+ naive, central memory, effector memory and terminal effector subsets. Regulatory T cells were assessed in 50 patients. An exploratory propensity-matched comparison was performed against a contemporaneous HLA-matched donor cohort.

**Results:**

Immune reconstitution followed a sequential pattern: NK cells recovered earliest (from day +21), B-cell recovery was delayed, and T cells expanded gradually, dominated by central and effector memory subsets, while naive and terminal effector subsets remained low through day +90. Grade II to IV acute GvHD occurred in 32.6% of patients, and transplant-related mortality (TRM) was 26.3% by day +100. No lymphocyte subset was significantly associated with acute GvHD, TRM or overall survival, and reconstitution kinetics were similar between myeloablative and reduced-intensity conditioning. In the propensity-matched comparison, day +100 TRM was higher in the haploidentical cohort, whereas acute GvHD and overall survival were comparable.

**Discussion:**

Early immune reconstitution after haploidentical transplantation with PTCy proceeds in a characteristic sequence, but quantitative recovery of circulating lymphocyte subsets alone did not predict acute GvHD or early mortality. Larger studies incorporating functional immune assays and time-to-event modelling are needed to clarify these relationships.

## 1 Introduction

Haplo-identical hematopoietic stem cell transplantation (Haplo-HCT) has emerged as an important therapeutic option for patients with hematological malignancies and non-malignant disorders lacking a suitable HLA-matched donor. The success of a haplo-HCT depends on several factors, including recipient and donor age, conditioning regimen, underlying comorbidities, transplant type, and HLA match. In addition, the choice of graft-versus-host disease (GvHD) prophylaxis strongly influences both engraftment kinetics and immune recovery. Despite significant advances in transplant strategies, GvHD remains a leading cause of morbidity and mortality and constitutes a major barrier to successful outcomes. It is typically associated with prolonged immunosuppression and delayed immune reconstitution, which increases susceptibility to bacterial, viral, and fungal infections, ultimately contributing to higher transplant-related mortality (1, 2). PTCy is now widely used as GvHD prophylaxis in allo-HCT across various donor types, particularly in the haploidentical setting. The use of PTCy in haplo-HCT has shown very encouraging outcomes, with survival rates comparable to those observed with HLA-matched HCT using standard GVHD prophylaxis, along with a significant reduction in chronic GvHD (3–6) and in excess non-relapse mortality (7, 8). Despite its widespread clinical use, the exact mechanisms by which PTCy reduces both acute and chronic GVHD are still not fully understood, and the distinct immune landscape associated with GVHD remains less explored.

Immune reconstitution is a dynamic, sequential process influenced by a range of factors, including donor-recipient HLA matching, graft source, conditioning regimen, GvHD prophylaxis, donor age, infection history, and others. The reconstitution of different immune subsets, after haplo-HCT, occurs at different time points. After conditioning, the patient undergoes an aplastic phase until neutrophils recover (9). Although neutrophil and platelet engraftment generally occurs within the initial weeks, lymphocyte reconstitution in adults is comparatively delayed. Among lymphocyte subsets, natural killer (NK) cells are usually the first to recover within the first few months’ post-transplant, followed by CD8+ and CD4+ T lymphocytes (9, 10). Timely reconstitution of donor-derived immune cells following haplo-HCT is essential for favorable outcomes, as many post-transplant complications arise from a dysregulated immune state.

The mechanisms underlying PTCy-mediated immune tolerance remain poorly understood. Current evidence suggests that PTCy selectively eliminates alloreactive T cells of both donor and host origin during the early post-transplant period, while relatively sparing regulatory T cells (Tregs) (5). This is supported by murine skin allograft models (11), in which cyclophosphamide administration shortly after transplant led to the elimination of donor-derived alloreactive T cell populations via an extrathymic mechanism (12, 13). Concurrently, Treg cells appear to be preferentially preserved, potentially due to elevated expression of aldehyde dehydrogenase enzymes, which confer resistance to cyclophosphamide-induced cytotoxicity (14).

In the present study, we prospectively analyzed immune reconstitution in a cohort of haplo-HCT recipients receiving PTCy-based GvHD prophylaxis, characterizing the kinetics of T cells, CD4+ and CD8+ T cells, NK cells, and B lymphocytes over the first 100 days after transplantation and exploring their association with early clinical outcomes, including acute GvHD and transplant-related mortality at Day +30 and Day +100. Given the descriptive nature and sample size of this single-center cohort, the outcome comparisons were regarded as exploratory rather than confirmatory.

In a sub-analysis of 50 patients, we also examined Treg recovery following transplantation to better understand the pattern of Treg reconstitution and its relationship with GvHD development and immune recovery in the post-transplant period.

## 2 Patients, materials, and methods

This prospective study included all consecutive patients who underwent haplo-HCT with PTCy-based GvHD prophylaxis in the Department of Haematology, Christian Medical College, Vellore, from October 2021 to December 2024. Patients were enrolled irrespective of the diagnosis after obtaining written informed consent/assent from patients or parents, respectively. The Institutional Review Board of the Christian Medical College, Vellore (IRB No 13919) approved this study. Patients undergoing a second transplant, those who underwent haplo-HCT with αβ T-cell depletion or had ECOG > 2 at the time of transplantation were excluded from the study. GvHD prophylaxis consists of Cy 50mg/kg/day on day 3 and day 4 (1 hour IV infusion), MMF from days 5 to 35 (15mg/kg per dose Q8H from day 5 with a maximum dose of 3g daily), and CsA (2.5mg/kg per dose Q12H from day 5 till day 90 for malignancies and longer for non-malignant conditions).

### 2.1 Comparator cohort and propensity-score matching

For the exploratory outcome comparison, a contemporaneous cohort of 177 patients who underwent HLA-matched allogeneic PBSC transplantation at the same center over the same period (February 2021 to December 2025) was used as the comparator group. These patients received grafts from HLA-matched related (n = 144) or matched unrelated (n = 33) donors, with GvHD prophylaxis based on a calcineurin inhibitor (cyclosporine or tacrolimus) combined with methotrexate; none received PTCy. The cohort comprised patients with malignant disease (n = 92, predominantly acute myeloid leukemia, myelodysplastic syndrome and chronic myeloid leukemia) and with non-malignant marrow failure (n = 85, severe aplastic anemia and Fanconi anemia); conditioning was predominantly fludarabine-based. The median age was 29 years (range, 1–65) and 73.4% were male. Because a proportion of PTCy patients had lymphoid malignancies or other non-malignant disorders (primarily primary immunodeficiencies and inherited metabolic disorders) with no counterpart in the comparator, matching was restricted to patients with complete baseline covariate data, yielding 63 pairs; the matched haploidentical arm comprised myeloid malignancy (47.6%), marrow failure (20.6%) and other diagnoses (31.7%), and no patient with lymphoid malignancy was included in either arm. The matching procedure and balance assessment are described under Statistical analysis. Cytomegalovirus (CMV) surveillance differed by donor type: haploidentical and matched-unrelated donor (MUD) recipients were monitored weekly, whereas matched-related (sibling) donor recipients were tested once, at day +28. This difference in surveillance frequency is relevant to the interpretation of viral infection rates and is addressed in the limitations.

### 2.2 Immunophenotyping

Serial peripheral blood (PB) samples were collected at predetermined time points: pre-conditioning, the third consecutive day of neutrophil engraftment (defined by absolute neutrophil count [ANC] recovery), and Days +21, +28, +60, and +90 post-HCT, for flow cytometric immunophenotyping. Multicolor flow cytometry was performed on a DxFLEX instrument (Beckman Coulter, USA) using a panel comprising: APC-anti-CD3, FITC-anti-CD4, BV786-anti-CD8, ECD-anti-CD64, PerCP-Cy5.5-anti-CD56, PE-Cy-anti-CD19, APC-H7-anti-CD45, BV421-anti-CD27, V510-anti-CD45RA, and BV605-anti-CD16. Data acquisition and analysis were performed using Kaluza software v2.2 (Beckman Coulter). Immunophenotyping was performed to enumerate major lymphocyte populations and their subsets across all timepoints, including T cells (CD45^+^CD3^+^), B cells (CD45^+^CD19^+^), and NK cells (CD45^+^CD3^–^CD16^+^CD56^+^CD64^–^). Within the T cell compartment, helper T cells (Th; CD3^+^CD4^+^CD8^–^) and cytotoxic T cells (Tc; CD3^+^CD8^+^CD4^–^) were further resolved into four differentiation stages based on CD27 and CD45RA expression: naïve (CD27^+^CD45RA^+^), central memory (CM; CD27^+^CD45RA^–^), effector memory (EM; CD27^–^CD45RA^–^), and terminal effector (TD; CD27^–^CD45RA^+^) subsets.

T regulatory cell (Treg) reconstitution was assessed separately using donor harvest samples and serial post-transplant recipient PB samples (as same as above). Tregs were identified as CD4^+^CD25^+^CD127^–^FOXP3^+^ cells using a dedicated panel comprising CD4-FITC (clone SK3), CD25-PE-Cy7 (clone 2A3), and CD127-Alexa Fluor 647 (clone hIL-7R-M21).

Absolute subset counts were derived by normalizing flow cytometric proportions to the concurrent WBC count at each respective time point and are expressed as cells/µl.

### 2.3 Clinical outcomes

Details on patient and transplant characteristics as well as outcomes were extracted from our electronic medical record database. Early post-transplant outcomes, including engraftment (neutrophil and platelet), graft rejection, acute GVHD, chimerism status, and survival, were documented in the clinical database. Neutrophil engraftment was defined as an absolute neutrophil count>500/mm3 for three consecutive days, while platelet engraftment was defined as a platelet count >20,000/mm3 without transfusion support for seven consecutive days. Complete chimerism was defined as having more than 95% of donor genetic marker patterns, and Mixed Chimerism was defined as the presence of more than 5% residual host chimerism. Rejection was >90% residual host chimerism in peripheral blood (15). GVHD was graded according to the Glucksberg criteria (16). In the present study, patients with Grade II-IV GvHD were categorized as acute GvHD, and those with Grade I GvHD, and those who did not develop GvHD were classified as the No GvHD group (Grade I GvHD is usually resolvable without systemic treatment and therefore included in the No GvHD group). Any deaths (non-relapse deaths) occurring within the first 100 days post-HCT were considered Transplant Related Mortality (TRM). TRM Day +30 was defined as deaths occurring within 30 days, and TRM Day +100 was defined as deaths occurring within 100 days post-HCT.

### 2.4 Statistical analysis

For the exploratory comparison with the matched-donor cohort, propensity scores were estimated by logistic regression on age, sex, disease category (myeloid, lymphoid or marrow failure), CD34 dose and the hematopoietic cell transplantation-specific comorbidity index (HCT-CI). Patients were matched 1:1 by nearest-neighbor matching on the logit of the propensity score (caliper 0.2 standard deviations), with exact matching on disease group (marrow failure, lymphoid malignancy). Myeloid malignancy and other diagnoses (e.g. primary immunodeficiencies, Thalassemia, mucopolysaccharidoses) were not exactly matched, so their distribution could differ between arms after matching, and balance was assessed using standardized mean differences (values below 0.1 indicating adequate balance). Binary outcomes were compared using the McNemar test and overall survival using time-to-event methods accounting for the matched design (a Cox model stratified on matched pair, and the log-rank test). An exploratory multivariable logistic regression for grade II–IV acute GvHD (including age, sex, CD34 dose and HCT-CI) was also performed; given the limited number of events relative to covariates, this model was interpreted as exploratory. This platform comparison was exploratory. Demographic parameters and pre-transplant characteristics were summarized using descriptive statistics. Differences in immune subset levels between patients who developed acute GvHD and those who did not, as well as between those with and without transplant-related mortality (TRM) at Day +30 and Day +100, were assessed using the Mann–Whitney U test. Univariate survival analysis was conducted to evaluate the association between immune reconstitution at each time point and transplant outcomes, including aGvHD, TRM, and overall survival (OS). Fisher’s exact test was performed to evaluate the association between infection status and GvHD. All statistical analyses were performed using GraphPad Prism v8.4.3 (GraphPad Software, La Jolla, CA, USA) and IBM SPSS Statistics v23 (IBM Corp., Chicago, IL, USA).

## 3 Results

### 3.1 Patient demographics and post-transplant outcomes

This study included a total of 95 patients who underwent haplo-HCT using PTCy-based GvHD prophylaxis (**Table 1**). The median age of this group was 20.5 years (range, 1–62 years), with 64 (67.4%) male patients, while females accounted for 32.6% of the total population. A larger number of patients underwent transplant for hematological malignancies (61.1%), whereas 38.9% of the transplants were for non-malignant disorders. Peripheral blood stem cells (PBSC) were used as the graft source in 90 (94.7%) patients, while bone marrow (BM) was used in only 5 (5.3%) patients. Several conditioning regimens were used in this cohort, reflecting its heterogeneity in diagnosis. The commonly used regimens included Fludarabine/Busulfan (Flu/Bu – 27.4%), Flu/Treosulfan (Flu/Treo -23.2%), Flu/Melphalan (Flu/Mel -18.9%), Flu/Cy (14.7%), and Cy/TMLI (13.7%). In contrast, other regimens such as Flu/TBI, Flu/Rituximab/Paclitaxel/Mito/Carbo/Thio were used less frequently. 59 (62.1%) patients received a myeloablative conditioning (MAC) regimen, followed by reduced-intensity conditioning (RIC) in 35 (36.8%) cases, rest were NMA conditioning.

**Table 1:** Patient demographics (n = 95).

Patient characteristics n =95	Median (Range/%)
Age	20.5 (1-62)
Sex	
Male	64 (67.4%)
Female	31 (32.6%)
Diagnosis	
Malignant	58 (61.1%)
Non-malignant	37 (38.9%)
Graft source	
PBSC	90 (94.7%)
BM	5 (5.3%)
HLA Disparity	
5//10, 6//12	51 (53.7%)
others	44 (46.3%)
Pre-transplant conditioning regimen	
MAC	59 (62.1%)
RIC	35 (36.8%)
NMA	1 (1.1%)
Flu/Bu	26 (27.4%)
Flu/Treo	22 (23.2%)
Flu/Mel	18 (18.9%)
Flu/Cy	14 (14.7%)
Cy/TMLI	13 (13.7%)
OTHERS	2 (2.1%)
GvHD Prophylaxis	
PTCy/CsA/MMF	95 (100%)

Categorical variables are displayed as n(%). Continuous variables are expressed as median (range). PBSC, Peripheral Blood Stem Cells; BM, Bone Marrow; MAC- Myeloablative condition; RIC, Reduced intensity conditioning; NMA, Non-myeloablive conditioning; CsA, Cyclosporine A; MMF, Mycophenolate mofetil.

Chimerism was assessed at day +28, +60 and +100. Because chimerism cannot be evaluated in patients who fail to engraft or die early, the 6 patients with primary graft failure or early death were excluded, and chimerism is reported for the 89 engrafted patients. At day +28, complete donor chimerism was present in 83 of 89 engrafted patients (93.3%), with mixed chimerism in 2 (2.2%) and graft rejection in 1 (1.1%); chimerism data were unavailable for 3 engrafted patients (3.4%). Mixed chimerism remained a small, persistent subset across later time points. Neutrophil engraftment was achieved in 89 (93.7%) patients, with a median time to engraftment of 18 days (range, 12–28 days). The remaining 6 patients had either primary engraftment failure (n = 4) or early death (n = 2). Platelet engraftment was observed in 80 patients (84.2%), with a median duration of 20 days (range, 8–68 days). Out of the total study population, acute GvHD occurred in 39 (41.1%) patients, while 50 (52.6%) patients did not develop acute GvHD, and 6 (6.3%) patients were not evaluable due to no engraftment and early death. Excluding patients with primary graft failure or early death (evaluable n = 89), any-grade acute GvHD occurred in 39 patients (43.8%). Grade 2–4 aGvHD was observed in 29 patients (32.6%) and grade 3–4 aGvHD in 13 patients (14.6%). The median time to GvHD occurrence was 33 (14–114) days post-transplant. Transplant-related mortality (TRM) was observed in 5 patients (5.3%) by day +30 and in 25 patients (26.3%) by day +100 post-transplant. At the time of analysis, 58.9% of patients were alive, while 41.1% had died (**Table 2**). Infection and transplant-associated complications were the major causes of death. Infection status is summarized in **Tables 3A** and **3B**.

**Table 2:** Post-transplant outcomes.

HCT outcomes	N (%)
Chimerism status	
D+28	
CC	85 (89.5%)
MC	3 (3.1%)
Rejection	2 (2.1%)
NA	5 (5.2%)
D+60	
CC	54 (56.8%)
MC	6 (6.3%)
Rejection	1 (1.1%)
NA	34 (35.8%)
D+100	
CC	46 (48.4%)
MC	5 (5.2%)
Rejection	1 (1.1%)
NA	43 (45.3%)
Neutrophil engraftment	
No.of patients engrafted	89 (93.7%)
NE	6 (6.3%)
Median days to engraftment	18 (12-28)
Platelet engraftment	
No. of patients engrafted	80 (84.2%)
NE	15
Median day to engraftment	20 (8-68)
Acute GvHD (Evaluable n= 89)	
No GvHD	50 (52.6%)
Yes	39 (41.1%)
NE	6 (6.3%)
Grade I	10 (11.5)
Grade II	16 (18.3)
Grade III	6 (6.8)
Grade IV	7 (8)
Grade 0-I	60 (67.4%)
Grade II-IV	29 (32.6)
Grade III-IV	13 (14.6%)
Median days to GvHD Occurrence	33 (14-114)
Alive status	
TRM D 30 (Yes/No)	5/90
TRM D100 (Yes/No)	25/70
Alive	56 (58.9%)
Dead	39 (41.1%)

**Table 3A:** Overall infection distribution.

Infection category	n	%
Total no. of infection cases	65	68.4
No documented infection	30	31.6
Viral only	19	20.0
Bacterial + Viral	12	12.6
Bacterial + Viral + Fungal	10	10.5
Bacterial only	8	8.4
Bacterial + Fungal	6	6.3
Viral + Fungal	5	5.3
Fungal only	5	5.3
**Total**	**95**	**100**

Categorical variables are displayed as n(%) and median in range. GVHD, Graft vs Host Disease; CC, Complete Chimerism; MC, Mixed Chimerism; TRM, treatment related mortality; NA, Not available; NE, Not evaluable.

Bold type denotes column headings and the total row.

**Table 3B:** Overall prevalence by pathogen class.

Infection category	Organism	n (%)
Bacterial infections (n = 36)	*Clostridioides difficile*	8 (22.2)
	*Enterococcus faecium*	6 (16.7)
	*Klebsiella pneumonia*	3 (8.3)
	*Pseudomonas aeruginosa*	3 (8.3)
	*Acinetobacter* spp.	3 (8.3)
	*Staphylococcus aureus*	2 (5.6)
	Carbapenem-resistant *Klebsiella* (CRO)	2 (5.6)
	Other bacterial pathogens*	9 (25.0)
Viral infections (n = 46)	Cytomegalovirus (CMV)	20 (43.5)
	BK virus	5 (10.9)
	CMV and BK virus co-infection	4 (8.7)
	Human enterovirus/rhinovirus	2 (4.3)
	Other viral infections	15 (32.6)
Fungal infections (n = 26)	Probable fungal pneumonia	10 (38.5)
	Possible fungal pneumonia	8 (30.7)
	*Candida* spp.	2 (7.7)
	*Aspergillus* spp.	1 (3.8)
	*Fusarium* spp.	1 (3.8)
	Fungal rhinosinusitis	1 (3.8)
	Other fungal infections	3 (11.5)

Infection categories represent documented infectious episodes occurring during the post-transplant period. Percentages were calculated using the total number of patients with each infection type as the denominator. As some patients experienced multiple infectious episodes and/or had more than one pathogen identified, percentages may exceed 100%.

A total of 50 patients were included in the Treg reconstitution sub-analysis, with a median age of 23 years (range, 2–54 years). The cohort was predominantly composed of patients with malignant disorders. Baseline demographic characteristics, transplant-related variables, engraftment outcomes, chimerism status, GvHD incidence, and survival outcomes were summarized in **Table 4**.

**Table 4:** Patient demographics and post-transplant outcome details (Sub-analysis Tregs).

Patient demographics (n = 50)	
Age	23 (2-54)
Sex	
Male	34 (68%)
Female	16 (32%)
Diagnosis	
Malignant	39 (78%)
Non-malignant	11 (22%)
AML	16(32%)
ALL	13(26%)
MDS	6(12%)
CML	1(2%)
AA	8(16%)
Lymphoma	3(6%)
Others	2(4%)
Conditioning regimen	
Flu/Bu	12 (24%)
Flu/Cy	9(18%)
Flu/Mel	8(16%)
Flu/Treo	6(12%)
Cy/TBI	2(4%)
Cy/TMLI	11(22%)
Flu/TBI OR Flu/TMLI	2(4%)
Neutrophil engraftment	
Day	16 (12-23)
No.of patients engrafted	43 (86%)
NE	2 (4%)
Platelet engraftment	
Day	19 (8-30)
No.of patients engrafted	40 (80%)
NE	2 (4%)
aGvHD	
Yes	19 (38%)
No	30 (60%)
NE	1 (2%)
Median onset of GvHD	38 (18 -115)
VOD	10 (20%)
Mucositis	39 (78%)
Chimerism status	
Chimerism D + 28 (CC/MC/Rejection)	47/2/1 (94%/4%/2%)
Chimerism D + 60 (CC/MC/No data)	44/2/4 (88%/4%/8%)
Chimerism D + 100(CC/MC/No data)	38/4/8 (76%/8%/16%)
Alive/dead status	
Alive	45 (90%)
Expired	5(10%)

Categorical variables are displayed as n(%) GVHD, Graft vs Host Disease; CC, Complete Chimerism; MC, Mixed Chimerism; AML, Acute myeloid leukemia; ALL, Acute lymphocytic leukemia; MDS, Myelodysplastic syndrome; CML, Chronic myeloid leukemia; AA, Aplastic anemia; TRM, treatment related mortality; VOD, Veno-occlusive disease; NE, Not evaluable.

### 3.2 Immune reconstitution pattern following post-transplant cyclophosphamide in haplo-HCT

Immune reconstitution followed a consistent sequence (**Figure 1**; **Supplementary Table S1**). Lymphocyte counts declined sharply after conditioning and subsequently recovered, with the absolute lymphocyte count reaching ≥1000 cells/µl in 33 of 68 evaluable patients by day +60. NK cells recovered early, starting from day +21 and peaking around day +60. T cells were low at day +21 and +28 and expanded mainly from day +60, dominated by central and effector memory subsets, while naïve and terminal effector subsets remained low. B-cell recovery was the most delayed, low through day +28 and increasing only at day +60 and +90. Interpatient variability was wide at every time point. Median absolute counts for all subsets at each time point are given in **Supplementary Table S1**.

**Figure 1:**
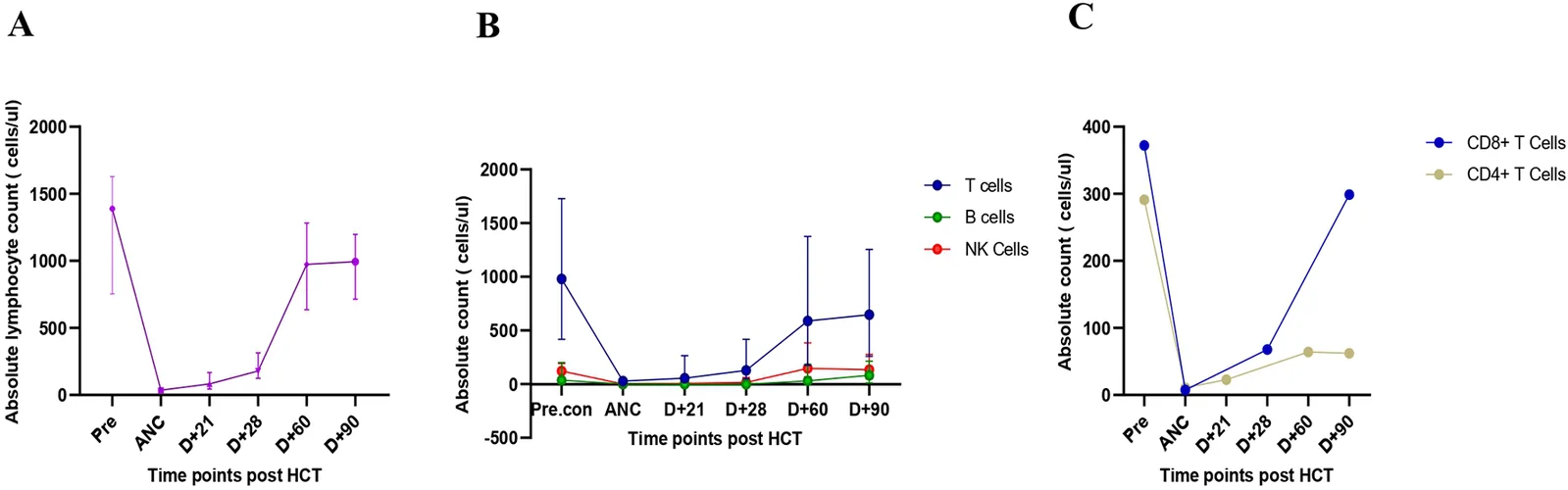
Reconstitution kinetics of lymphocyte subsets following haploidentical PBSCT. Median absolute counts (cells/μl) of each lymphocyte subset were plotted over time from Pre to Day+90 post transplantation. **(A)** Total lymphocytes, **(B)** T, B and NK cells. **(C)** CD4^+^ T cells and CD8+ T cells.

Infectious complications were common during the post-transplant period in this cohort. Overall, 31.6% of patients had no documented infections, while the remaining 68.4% patients experienced at least one infectious episode. Viral infections represented the most frequent pathogen class, occurring in 48.4% of patients, followed by bacterial infections in 37.9% and fungal infections in 27.4% of the cohort. Isolated viral infections were the most commonly observed infection pattern (20%), whereas mixed infections involving multiple pathogen classes were also observed, including combined bacterial and viral infections (12.6%) and concurrent bacterial, viral, and fungal infections (10.5%) (**Tables 3A**, **B**).

Within the T-cell compartment (**Figure 2**; **Supplementary Table S1**), both CD4+ and CD8+ subsets were low early and recovered mainly from day +60. In both lineages, central and effector memory subsets recovered fastest, whereas naïve and terminal effector subsets remained low relative to pre-transplant levels throughout the first 100 days, indicating memory-dominated early reconstitution.

**Figure 2:**
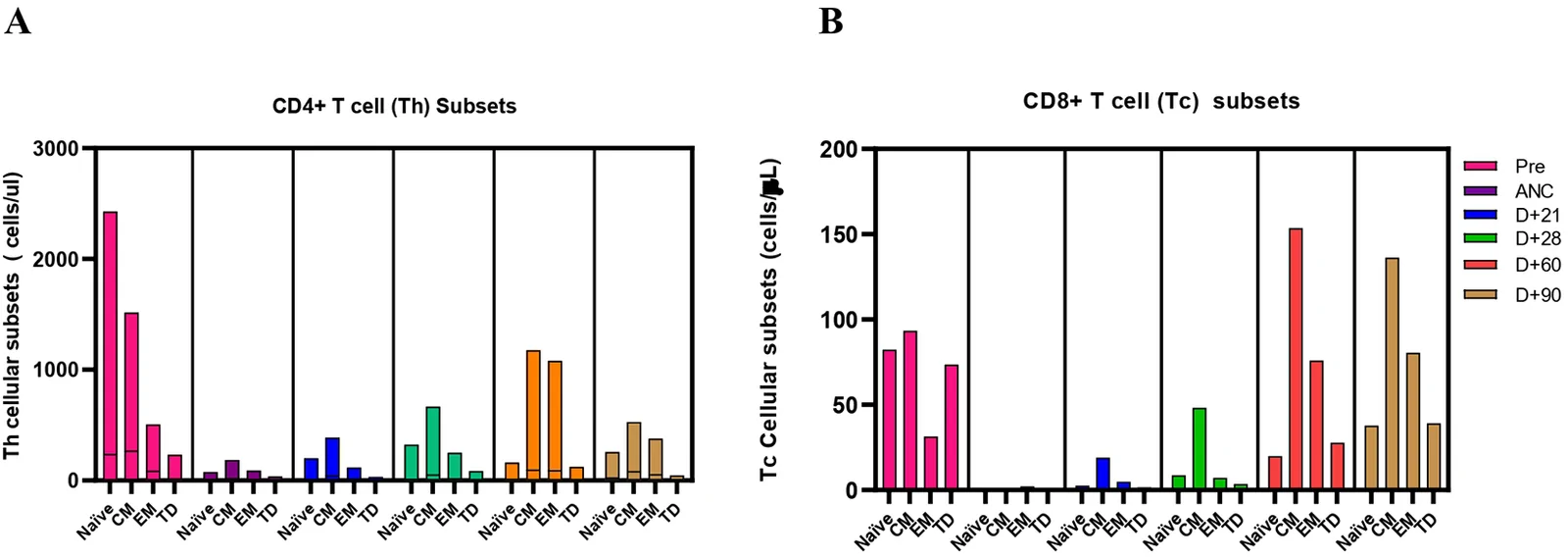
Immune reconstitution kinetics of **(A)** CD4+ and **(B)** CD8+ T cell subsets. Each bar indicates the corresponding cellular subset at appropriate post-transplant time points. CM, Central memory T cells; EM, Effector memory T cells; TD, Terminal effector T cells.

### 3.3 Comparison of Immune reconstitution and post-transplant outcomes

We next examined whether immune reconstitution was associated with acute GvHD, comparing engrafted patients with (grade II–IV) and without (grade 0–I) aGvHD (**Figure 3**; **Supplementary Table S2**). Patients who developed grade II–IV aGvHD showed numerically higher central memory T-cell counts at engraftment — CD8+ CM (p = 0.06) and CD4+ CM (p = 0.057) — but no subset reached statistical significance (**Figure 4**). For severe (grade III–IV) aGvHD, naïve CD4+, CD4+ terminal effector and CD8+ effector memory T cells differed only at day +28 (**Supplementary Table S4**). On univariate analysis, no immune subset or clinical variable was significantly associated with GvHD. Because acute GvHD occurred early (median onset day +33), subset measurements at day +60 and +90 were obtained after aGvHD onset in most affected patients; associations at these time points therefore reflect the consequences of aGvHD and its treatment rather than predictors, and only measurements at engraftment and day +28 can be regarded as potentially predictive. An exploratory multivariable logistic regression for grade II–IV aGvHD included clinical covariates only (age, sex, CD34 dose and HCT-CI); immune subsets were not entered, owing to the limited number of events. Only female sex was independently associated (p = 0.045). Of the 29 patients with grade II–IV aGvHD, one had missing HCT-CI data and was excluded, leaving 28 events across four covariates (approximately 7 events per variable); the model is therefore exploratory and should be interpreted with caution.

**Figure 3:**
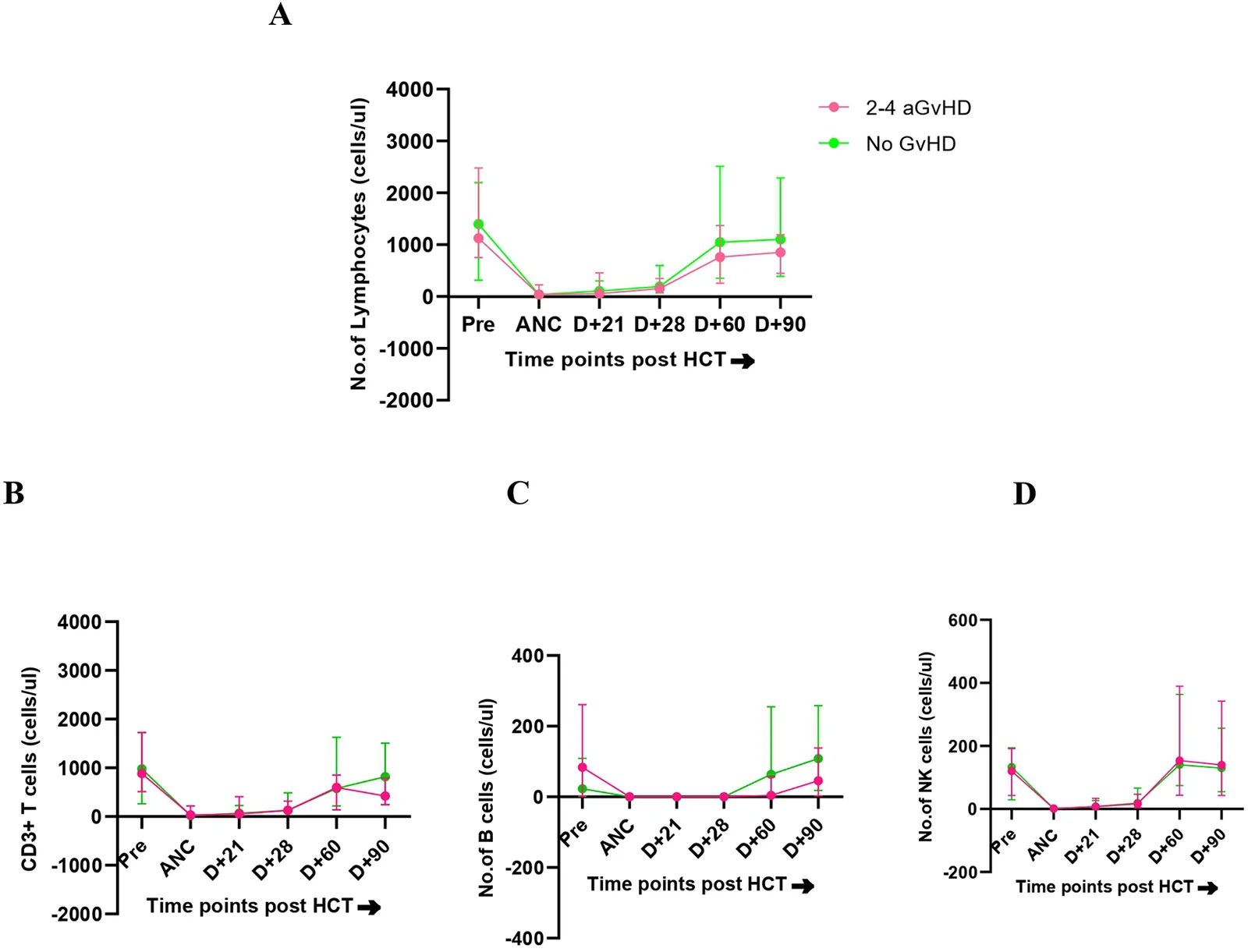
Comparison of immune reconstitution pattern and grade 2-4aGvHD (Median and interquartile range are illustrated). **(A)** Lymphocytes, **(B)** CD3+ T cells, **(C)** B cells and **(D)** NK cells.

**Figure 4:**
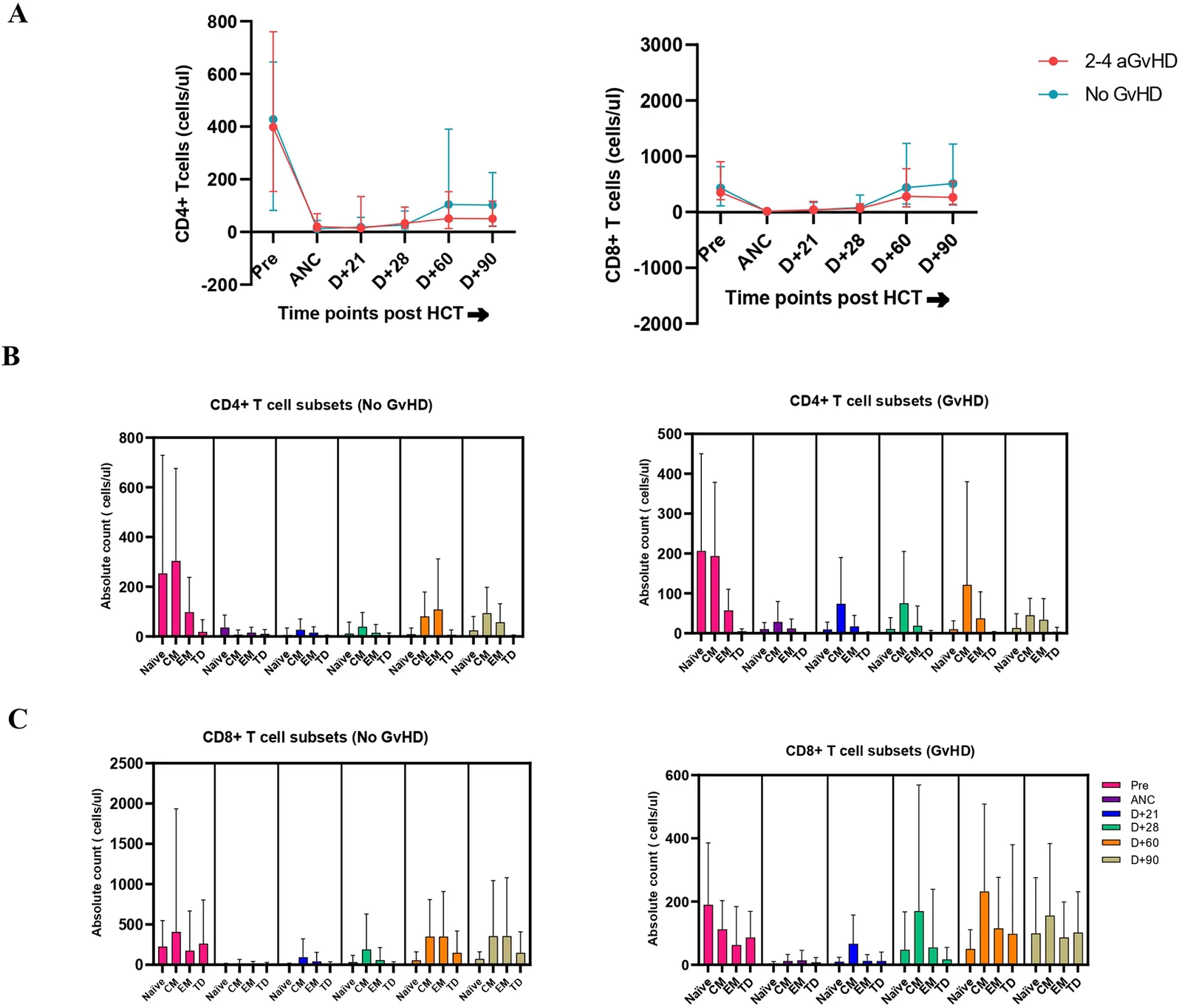
Comparison of immune reconstitution pattern and grade 2-4aGvHD. **(A)** No difference in the reconstitution pattern between patients with and without acute GvHD. **(B)** CD4+ T cells, **(C)** CD8+ T cells. CD8^+^ CM T-cell levels were higher in patients who developed grade II–IV acute GvHD at D + 28 but were not statistically significant (p = 0.06).

We further evaluated whether the occurrence of grade II–IV aGvHD was associated with post-transplant infectious complications. Infectious events were categorized into viral, bacterial, and fungal infections (**Table 3A**). Fisher’s exact test was performed to assess the association between the development of grade II–IV aGvHD and infection status. No statistically significant association was observed between the occurrence of acute GvHD and overall infectious complications. Infections occurred early after transplantation (median onset day +10, range 1–34), and all documented infections had occurred within day +60. In an exploratory analysis, patients who developed infection showed lower reconstitution of several lymphocyte subsets at day +60, most notably total B cells (median 17.3 versus 136.9 cells/µl, p = 0.006) and CD4+ T-cell subsets. Because infections preceded these measurements, this pattern reflects reduced immune recovery following infection and its treatment rather than a predictor of infection; when subset counts measured before infection onset were compared with those of never-infected patients, no subset predicted subsequent infection. These comparisons were exploratory and unadjusted for multiple testing.

### 3.4 Effect of conditioning intensity on immune reconstitution

Because conditioning intensity is known to influence post-transplant immune recovery, we compared lymphocyte reconstitution kinetics between patients receiving myeloablative conditioning (MAC) and reduced-intensity conditioning (RIC). The overall patterns of immune reconstitution were similar in both groups across all evaluated time points, including ANC recovery and days +21, +28, +60, and +90 post-transplant. No consistent differences in absolute lymphocyte counts were observed between MAC and RIC recipients, suggesting that conditioning intensity did not influence the immune reconstitution observed in this cohort (**Supplementary Figure S1A**).

### 3.5 T regulatory cell reconstitution following haplo-HCT with PTCy (Tregs, N = 50)

Immune reconstitution was evaluated at serial post-transplant time points from ANC recovery, at D + 28, D + 60, and D + 90 post-transplant in 50 patients undergoing haplo-HCT with PTCy-based GvHD prophylaxis. T cells were defined as CD3+ T cells, CD3+CD4+ T cells, and CD3+CD4+CD25+CD127- Tregs. Donor reference ranges are represented by the shaded regions in the lower panels (**Figure 5A**). A progressive increase in total T-cell and CD4+ T-cell counts was observed over time following transplantation. Similar to the above observation, Total CD3+ T-cell counts demonstrated marked expansion between day +28 and day +90, approaching donor reference levels by day +90. Similarly, CD4+ T-cell recovery remained delayed during the early post-transplant phase but gradually increased at later time points, with substantial recovery evident by day +60 and day +90 (**Figure 5B**; **Supplementary Table S5**).

**Figure 5:**
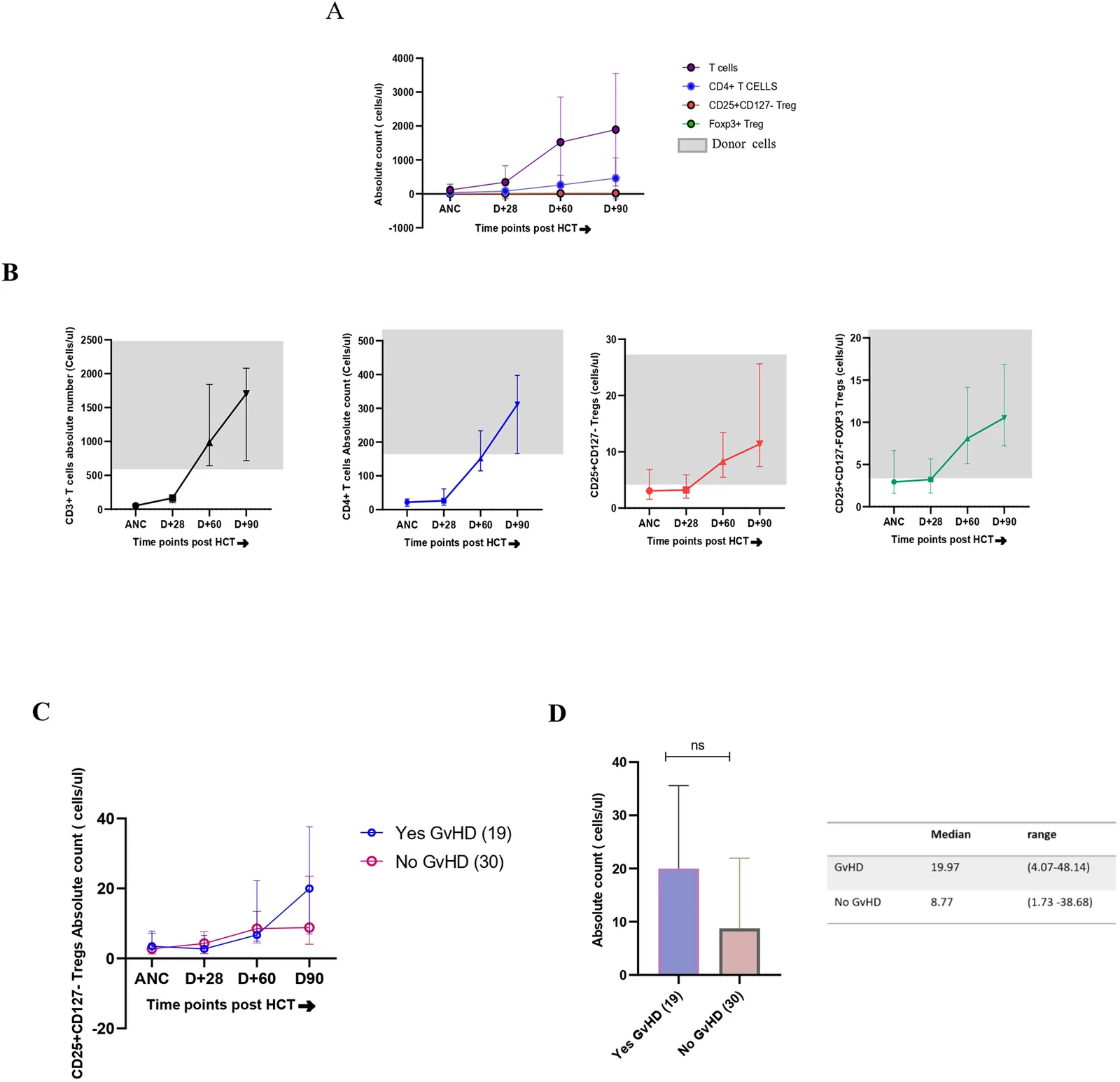
The immune reconstitution pattern for an individual cell subset over time. The gray area represents the donor median range (cells/ul). Median and interquartile range are represented. **(A, B)** T cells, CD4+ T cells, Tregs. **(C)** Difference between the Tregs immune reconstitution pattern of patients with and without aGvHD. **(D)** Mann-Whitney U test for D + 90 Tregs between with and without aGvHD. Median and range are represented, but there is no significant difference (p= 0.057).

Tregs demonstrated progressive recovery during the post-transplant period. Tregs remained relatively low during the initial post-transplant phase but showed gradual expansion beginning around day +60, with sustained recovery observed up to day +90. Although Treg counts remained below donor reference levels in several patients, the observed upward trend suggests gradual restoration of immune regulatory mechanisms over time. The recovery of Treg cells seemed to follow the overall trend of CD4+ T-cell reconstitution. This supports the idea that immune tolerance after PTCy-based haplo-HCT may partly come from preserving and expanding Treg cell populations. Overall, this finding shows the progressive reconstitution after haplo-HCT, characterized by delayed but progressive recovery of both CD4+ T cells and Treg subsets during the first 100 days post-transplant.

### 3.6 Comparison of immune reconstitution of Tregs and post-transplant outcomes

There was a total of 19 (38%) patients with various grades of aGvHD, with a median GvHD onset day of 38 days (18–115). 29 (60%) patients had no aGvHD, and 2 (4%) were not evaluable due to no engraftment. On comparing the immune reconstitution pattern between the patients who developed aGvHD v/s those who did not develop GvHD, it was observed that the total number of Tregs was not significantly different between patients with and without aGvHD (**Figure 5C**; **Supplementary Tables S5**, **S6**). Both patients with GvHD and without GvHD followed a similar reconstitution pattern for Tregs, with a slight difference at D + 90. We then performed a Mann-Whitney U test to assess the Treg pattern at D + 90. There was no significant difference in the reconstitution pattern of Tregs on D + 90 between patients with {19.97 (4.074 – 48.15), n = 19, p= 0.057} and without aGvHD {8.77 (1.73 – 38.68), n= 30}.

### 3.7 Effect of conditioning intensity on immune reconstitution (Treg Sub-analysis)

We compared CD3+ T cell reconstitution pattern between patients receiving myeloablative conditioning (MAC) and reduced-intensity conditioning (RIC) regimens (**Supplementary Figure S1B**) in this cohort. Both groups showed a similar pattern of T cell reconstitution after transplantation. This pattern included significant lymphodepletion during the early post-transplant period, followed by gradual recovery starting from day +28. We observed a noticeable increase in T cells at days +60 and +90 in both groups, indicating ongoing immune recovery. Similar reconstitution patterns were observed between MAC and RIC recipients across all evaluated time points, suggesting that conditioning intensity did not substantially influence early immune reconstitution in this cohort.

### 3.8 Exploratory comparison with matched-donor transplantation

To place these findings in context, early outcomes of the study cohort were compared with a contemporaneous cohort of HLA-matched related and unrelated donor transplants performed at the same center, using propensity-score matching (see Methods). Sixty-three patient pairs were matched. Patients with lymphoid malignancy in the study cohort had no comparator counterpart and were not matched. After matching, age, sex and the proportion with marrow failure were well balanced (SMD ≤0.07; **Table 6**). Balance was not achieved for all covariates: myeloid disease remained more frequent in the matched-donor cohort (73.0% vs 47.6%; SMD 0.54), whereas other diagnoses (primary immunodeficiencies, Thalassemia, mucopolysaccharidoses) were more frequent in the haploidentical arm (31.7% vs 6.3%; SMD 0.68). CD34 dose (SMD 0.20) and HCT-CI (SMD 0.12) exceeded the prespecified threshold of 0.1 (**Table 5**; **Supplementary Figure S2**). The comparison is therefore exploratory and subject to residual confounding by disease group and baseline risk.

**Table 5:** Post-matching baseline characteristics (63 matched pairs).

Covariate	Haplo-PTCy (n=63)	Matched-donor (n=63)	|SMD|
Age (years)	21	22	0.00
Female sex	33.3%	30.2%	0.07
CD34 dose (×10^6^/kg)	10.0	10.0	**0.20**
HCT-CI	0	0	**0.12**
Myeloid malignancy	47.6%	73.0%	**0.54**
Marrow failure	20.6%	20.6%	0.000
Lymphoid malignancy	0.0%	0.0%	0.000
Other	31.7%	6.3%	**0.68**

Post-matching baseline characteristics in the propensity-matched cohort (63 pairs) comparing haplo-HCT with PTCy versus matched-donor transplantation. A relatively greater imbalance was observed for myeloid disease (SMD 0.54). Continuous variables are shown as medians and categorical variables as percentages. Absolute standardized mean differences (SMD) were calculated with the pooled standard deviation; SMD <0.1 indicates adequate balance. Residual imbalance (SMD ≥0.1) remained for Other diagnoses (PID, Thal etc.), myeloid malignancy, CD34 dose and HCT-CI. Bold values indicate residual covariate imbalance after matching (|SMD| ≥ 0.10.

In the matched cohort, day +100 transplant-related mortality was significantly higher in the haploidentical cohort than in matched-donor recipients (28.6% versus 11.1%; McNemar p = 0.035). Grade II–IV acute GvHD, overall mortality and overall survival did not differ significantly between the two platforms (**Table 6**). Because the two platforms differ simultaneously in donor HLA disparity and GvHD prophylaxis, and because the matched sample was limited in size, these comparisons are exploratory and are interpreted further in the discussion.

**Table 6:** Propensity-matched comparison of transplant outcomes between haploidentical PTCy and matched-donor HCT recipients.

Outcome	Haplo-PTCy (n=63)	Matched-donor (n=63)	p
Transplant-related mortality, day +100	28.6% (18/63)	11.1% (7/63)	0.035
Grade II–IV acute GvHD	40.7% (24/59)	54.5% (30/55)	0.19
Death, any cause	42.9% (27/63)	34.9% (22/63)	0.46
Overall survival, median (months)	24.5	19.4	0.15

Early outcomes in the propensity-matched cohort (63 pairs) comparing haploidentical transplantation with post-transplant cyclophosphamide versus contemporaneous HLA-matched donor transplantation. Comparisons are exploratory. Binary outcomes were compared using the McNemar test; overall survival was compared using the log-rank test (p = 0.15), and a Cox model stratified on matched pair gave a hazard ratio of 1.16 (95% CI 0.63–2.14, p = 0.64).

## 4 Discussion

In this prospective study, we characterized early immune reconstitution following haplo-HCT with PTCy-based GvHD prophylaxis and evaluated its association with major early post-transplant outcomes. Our findings demonstrate that immune recovery after haplo-HCT with PTCy follows a distinct pattern, characterized by early natural killer (NK)-cell recovery, delayed B-cell reconstitution, and gradual expansion of T-cell subsets over the first 100 days post-transplant. Despite trends toward higher central memory T-cell counts in patients who developed acute GvHD (aGvHD), early immune reconstitution patterns were largely comparable between patients with and without aGvHD.

In this study, NK cells represented the earliest recovering lymphocyte subset, with progressive recovery observed from day +21 onwards. This observation is consistent with previous reports demonstrating that NK-cell reconstitution occurs rapidly after transplantation because NK cells recover mainly through peripheral expansion rather than thymic-dependent maturation (17, 18). Early NK-cell recovery has also been associated with protection against relapse and infections in several transplant settings (19). In contrast, B-cell recovery remained delayed during the early post-transplant phase. Delayed B-cell reconstitution after haplo-HCT has been well described previously (20) and may reflect impaired bone marrow microenvironment recovery and the prolonged effects of immunosuppressive therapy. Similarly, both CD4+ and CD8+ T-cell compartments demonstrated profound suppression during the earliest assessed post-transplant time points (day +21 and day +28), followed by gradual recovery thereafter. Among T-cell subsets, central memory (CM) and effector memory (EM) populations recovered earlier and more robustly than naïve T-cell subsets. This preferential recovery of antigen-experienced memory T cells likely reflects peripheral expansion of mature donor-derived lymphocytes rather than *de novo* thymopoiesis, which is generally delayed in adult transplant recipients. Naïve T-cell regeneration depends largely on thymic recovery, which may be impaired by conditioning regimens, infections, and ongoing immunosuppression. In contrast, memory T-cell subsets are capable of more rapid peripheral proliferation and therefore dominate the early immune landscape after transplantation. The predominance of memory T-cell recovery observed in our cohort may also partly explain the relatively preserved pathogen-specific immunity despite delayed global immune reconstitution.

One of the major objectives of this study was to evaluate the relationship between immune reconstitution and the development of aGvHD. Although patients who developed grade II–IV aGvHD demonstrated higher CD4+ and CD8+ central memory T-cell counts at the time of neutrophil engraftment, these differences did not reach statistical significance, and it is possible that subtle relationships may not have been detected because of the limited sample size and the heterogeneous nature of the study population. When the analysis was restricted to severe (grade III–IV) acute GvHD, two subsets differed nominally at Day +28, with terminal effector CD4+ T cells were lower and effector memory CD8+ T cells differed between groups (p = 0.019 and p = 0.021, respectively; **Supplementary Table S4**). However, these comparisons were exploratory, involved multiple immune subsets across several timepoints, and were not adjusted for multiple testing; they should therefore be regarded as hypothesis-generating rather than as established predictors of GvHD. Therefore, larger adequately powered studies together with clinical covariates in multivariable time-to-event models may help identify clinically relevant associations that were not apparent in the present analysis. Overall, immune reconstitution kinetics remained similar between patients with and without aGvHD. These findings suggest that early quantitative recovery of major lymphocyte subsets alone may not be sufficient to predict the occurrence of clinically significant aGvHD following PTCy-based haplo-HCT.

Previous studies evaluating immune reconstitution after haplo-HCT with PTCy have reported heterogeneous findings regarding the association between lymphocyte recovery and GvHD risk (19–21). Some studies have demonstrated associations between delayed CD4+ T-cell recovery and increased infectious complications or mortality, whereas others have suggested that rapid effector T-cell expansion may predispose to GvHD (22, 23). However, the predictive value of individual immune subsets remains inconsistent across studies, likely due to differences in transplant platforms, conditioning regimens, graft sources, timing of immune monitoring, and methods of immune phenotyping. The absence of strong immune predictors of aGvHD in our cohort may therefore reflect the complex and multifactorial biology underlying GvHD pathogenesis.

The high burden of infectious complications observed in our cohort further indicates the clinical relevance of immune reconstitution following PTCy-based haplo-HCT. Viral infections represented the predominant infectious complication, followed by bacterial and fungal infections, consistent with previous reports demonstrating delayed adaptive immune recovery and impaired antiviral immunity in the early post-transplant period after PTCy exposure (24, 25). Despite observing broadly comparable quantitative immune recovery kinetics between patients with and without acute GvHD, these findings do not necessarily imply equivalent functional immune competence. In addition, absolute immune subset recovery may not fully reflect qualitative immune dysfunction, impaired antigen-specific responses, or tissue-level immune dysregulation. Future studies integrating longitudinal immune phenotyping with detailed pathogen-specific infectious outcomes, particularly CMV and BK reactivation, may help clarify the relationship between immune recovery patterns and functional post-transplant immunity following PTCy-based haplo-HCT.

Corticosteroid therapy is an important confounder of the relationship between acute GvHD and immune reconstitution. In this cohort, corticosteroid exposure was almost entirely restricted to patients with grade II–IV acute GvHD: of the 29 patients with grade II–IV aGvHD, 27 received corticosteroids and 2 did not, whereas only 2 of the 59 patients without steroid exposure developed grade II–IV aGvHD, reflecting the use of steroids as first-line GvHD treatment. Steroid-exposed patients showed significantly lower late T-cell reconstitution, including day +90 CD4+ (median 39.2 versus 124.8 cells/µl, p = 0.007) and total T cells (374 versus 936 cells/µl, p = 0.011), consistent with the known lympholytic effect of corticosteroids on CD4+ T cells. Because steroid use and grade II–IV aGvHD overlapped almost completely, their effects on immune recovery could not be separated in this cohort; the lower late CD4+ recovery observed in patients with aGvHD may therefore be attributable to corticosteroid treatment rather than to GvHD itself. Quantitative circulating lymphocyte recovery may also not fully reflect functional immune competence at the tissue level.

We also observed broadly similar immune reconstitution kinetics between malignant and non-malignant disorders, although patients with non-malignant diseases showed a tendency toward faster recovery of immune subsets by day +90. Nevertheless, the overall patterns of immune recovery remained comparable between the two groups, suggesting that PTCy-based haplo-HCT supports consistent immune reconstitution across diverse disease indications. Another factor to incorporate when interpreting immune reconstitution studies is the intensity of the conditioning regimen. Myeloablative conditioning has traditionally been associated with deeper lymphodepletion compared with reduced-intensity approaches. However, in our cohort, lymphocyte recovery kinetics were similar between MAC and RIC recipients throughout the first 100 days following transplantation. These findings suggest that the differences observed between the aGvHD and non-aGvHD groups were unlikely to be primarily driven by conditioning regimen intensity. The heterogeneous nature of our study population, including differences in underlying diagnoses, patient age, and conditioning intensity, may have contributed to variability in immune recovery patterns. Rather, post-transplant immune recovery in the setting of PTCy-based haplo-HCT may be more strongly influenced by mechanisms such as peripheral expansion of donor-derived lymphocytes and recovery of memory T-cell populations than by the initial conditioning intensity itself. Larger studies with stratified analyses are needed to confirm these observations.

In the Treg sub-analysis, we observed gradual recovery of CD4+CD25+CD127−FOXP3+ Tregs beginning around day +60 post-transplant. Although Treg counts remained below donor reference ranges in several patients, the progressive increase over time supports the hypothesis that regulatory T cells may contribute to immune tolerance after PTCy-based haplo-HCT. However, we measured Treg numbers only; Treg counts were not significantly associated with aGvHD, and Treg suppressive function was not assessed. Any contribution of Tregs to tolerance therefore remains speculative, and Treg function rather than number may be the relevant determinant. These observations are consistent with experimental and clinical studies suggesting that Tregs exhibit relative resistance to cyclophosphamide-mediated cytotoxicity, potentially due to high aldehyde dehydrogenase expression (14). Despite the proposed central role of Tregs in mediating immune tolerance after PTCy, we did not observe significant differences in Treg reconstitution between patients with and without aGvHD. This finding may suggest that quantitative recovery of circulating Tregs alone does not fully capture the complex immunoregulatory mechanisms underlying GvHD prevention. Functional characteristics of Tregs, tissue-specific immune responses, cytokine profiles, and interactions with antigen-presenting cells may play equally important roles in determining clinical outcomes. Additionally, the relatively small sample size of the Treg cohort may have limited the power to detect clinically relevant differences.

In an exploratory analysis, we compared early outcomes in our PTCy-based haplo-HCT cohort with a contemporaneous cohort of HLA-matched related and unrelated donor transplants receiving calcineurin inhibitor-based prophylaxis at the same center, using propensity-score matching on age, sex, disease category, CD34 dose and HCT-CI (63 matched pairs). Day +100 transplant-related mortality was significantly higher in the haploidentical cohort (28.6% versus 11.1%), whereas acute GvHD did not differ significantly, and overall survival did not differ between the two groups (stratified Cox hazard ratio 1.16, 95% CI 0.63–2.14, p = 0.64; log-rank p = 0.15). This higher early mortality is consistent with the greater risks generally associated with HLA-mismatched transplantation, but the comparison must be interpreted cautiously. The two groups differ simultaneously in donor HLA disparity and in GvHD prophylaxis, so the higher transplant-related mortality cannot be attributed to PTCy as opposed to donor mismatch, and may reflect the mismatched-donor setting itself. Matching left residual imbalance in disease group and baseline covariates, and the matched sample remained modest in size, so these findings are exploratory and hypothesis-generating. A dedicated, adequately powered comparison, ideally against a non-PTCy haploidentical cohort, is needed to evaluate the specific contribution of PTCy. Viral infection was more frequent in the haploidentical cohort than in the comparator overall (48.4% versus 23.2%; []**Table 5**), but this difference is confounded by CMV surveillance frequency. When stratified by monitoring intensity, viral infection rates were comparable between the two weekly-monitored groups — haploidentical (48.4%) and matched-unrelated donor recipients (51.9%) — and markedly lower in matched-related (sibling) recipients (16.5%), who were tested only once at day +28 (weekly-monitored versus single-test comparator, p = 0.0003). The apparent excess of viral infection after haploidentical transplantation therefore reflects more frequent CMV surveillance rather than a true difference in infection rates. Bacterial and fungal infection rates, which are less dependent on scheduled surveillance, did not differ significantly between platforms.

This study has several strengths, including its prospective design with serial immune monitoring across predefined time points during the early post-transplant period, and detailed immunophenotyping of both conventional T-cell subsets and regulatory T cells. Several limitations should be acknowledged. First, the reconstitution patterns we describe confirm previous findings in a real-world cohort rather than extending mechanistic understanding. Second, it was conducted at a single center in a relatively small cohort, and the subgroup analyses (regulatory T cells, severe GvHD, and the propensity-matched comparison) were underpowered; a non-significant result in these analyses does not establish the absence of an effect. Third, the cohort was heterogeneous in diagnosis, conditioning regimen, and age, which limits the generalizability of subgroup findings. Fourth, and most importantly, we evaluated quantitative immune reconstitution — absolute subset counts and phenotype — and not functional immune competence. GvHD, infection susceptibility and mortality are determined substantially by immune functionality, including T-cell activation and antigen-specific responses, NK-cell cytotoxicity, cytokine production, and regulatory balance, none of which our data capture; quantitative recovery therefore cannot be equated with functional immunity, and our findings should be read strictly as describing quantitative reconstitution. For the same reason, our regulatory T-cell analysis assessed Treg numbers only, without suppressive-function or subset-heterogeneity data; the gradual Treg recovery we observed is descriptive, and Treg function rather than number may be the determinant of GvHD protection. Fifth, the immune–outcome analyses were largely exploratory: comparisons across multiple subsets and time points were not corrected for multiple testing, and the exploratory multivariable model for aGvHD (clinical covariates only) had few events per covariate, so nominal associations should be regarded as hypothesis-generating. Sixth, the propensity-matched comparison with matched-donor transplants is confounded by design, as the two groups differ simultaneously in donor HLA disparity and in GvHD prophylaxis; observed differences therefore cannot be attributed to post-transplant cyclophosphamide specifically, and residual confounding by unmeasured factors is likely; in addition, balance was not achieved for disease group (Others SMD 0.68, myeloid malignancy 0.54), CD34 dose (0.20) or HCT-CI (0.12). Seventh, infections were recorded as documented events without adjudication, onset timing, or standardized surveillance; differential monitoring (particularly for cytomegalovirus) may have influenced detected viral rates, and infection data were incomplete for part of the comparator cohort. Finally, follow-up was limited to day +100 and cannot address chronic GvHD, relapse, late infection, or long-term survival, and some baseline covariates were unavailable.

In conclusion, our study demonstrates that immune reconstitution after haplo-HCT with PTCy follows a sequential pattern with early NK-cell recovery, delayed B-cell recovery, and progressive expansion of memory T-cell subsets during the first 100 days post-transplant. Early immune recovery patterns were broadly comparable between patients with and without aGvHD, suggesting that quantitative lymphocyte reconstitution alone may not adequately predict GvHD development in the setting of PTCy-based prophylaxis. The gradual recovery of Tregs observed in our cohort further supports the concept that immune tolerance after PTCy may involve preservation and expansion of regulatory immune pathways. Future studies integrating functional immune assays, cytokine profiling approaches may provide deeper insights into the mechanisms linking immune reconstitution and clinical outcomes after haplo-HCT.

## Data Availability

The original contributions presented in the study are included in the article/**Supplementary Material**. Further inquiries can be directed to the corresponding author.
