# Supplementary material for: Kinetics of early immune reconstitution and its exploratory association with post-transplant outcomes in patients undergoing haploidentical HCT with post-transplant cyclophosphamide-based GvHD prophylaxis
Source: Front Immunol. 2026 Oct 26;17:1996185. doi: 10.3389/fimmu.2026.1996185 (PMC13745543; doi:10.3389/fimmu.2026.1996185)
Supplement: Supplementary file 1 [file DataSheet1.docx]

Supplementary Table S1:

|  | **PRE** | **ANC** | **D+21** | **D+28** | **D+60** | **D+90** |
| --- | --- | --- | --- | --- | --- | --- |
| **Lymphocytes** | 1389 | 35.54 | 82.87 | 179.5 | 974.4 | 993.6 |
|  | 93 - 15778 | 1 - 1299 | 4.7 - 2256 | 6.5 - 5773 | 5.5 - 8504 | 8.9 - 16191 |
| **T Cells** | 979.4 | 28.3 | 55.4 | 129.1 | 589.3 | 646.7 |
|  | 63.5 - 14929 | 0.9 - 840.6 | 2.8 - 2246 | 6.0 - 4699 | 2.5 - 6220 | 5.1 - 12483 |
| **B Cells** | 36.6 | 0.04 | 0 | 0 | 25.6 | 63.6 |
|  | 0 - 1200 | 0 - 81.6 | 0 - 174.3 | 0 - 48.1 | 0 - 1359 | 0 - 751.8 |
| **NK Cells** | 122.5 | 1.06 | 8 | 16.2 | 147.1 | 134.7 |
|  | 2.88 - 815 | 0 - 456.9 | 0 - 998.6 | 0.2 - 1563 | 1.5 - 3182 | 3.4 - 1697 |
| **CD3+CD4+ Tcells (Th cells)** | 418 | 12.3 | 16.3 | 26.6 | 78.5 | 80.3 |
|  | 9.3 - 3908 | 0.08 - 319.3 | 2.2 - 546.1 | 0 - 1094 | 0.2 - 1427 | 1.0 - 812.3 |
| **CD3+CD8+ T cells (Tc cells)** | 424.9 | 7.895 | 40.2 | 70.7 | 382.8 | 328.8 |
|  | 22.2 - 12284 | 0.02 - 507.7 | 0 - 2052 | 0.1 - 4340 | 0 - 4549 | 4.0 - 9631 |
| **CD4+ T Cell Subsets** | | | | | | |
| **NAÏVE** | 99.8 | 1.3 | 0.8 | 0.8 | 2.9 | 3.2 |
|  | 0.1 - 2427 | 0 - 7.27 | 0 - 200.5 | 0 - 323.4 | 0 - 162 | 0 - 258.1 |
| **Central Memory** | 132.4 | 4.9 | 8.8 | 19.4 | 48.9 | 54.7 |
|  | 3.8 - 1517 | 0.03 - 184.9 | 0.6 - 387.7 | 0 - 666.5 | 0 - 1177 | 0 - 527.9 |
| **Effector memory** | 43.5 | 4.7 | 4.9 | 4.6 | 24.3 | 27 |
|  | 0.5 - 504.8 | 0.06 - 90.7 | 0 - 116.7 | 0 - 251.6 | 0 - 1081 | 0.7 -379.4 |
| **Terminal Effector** | 2.661 | 0.04 | 0 | 0 | 0.955 | 1.148 |
|  | 0 - 233.2 | 0 - 37.8 | 0 - 31.9 | 0 - 86.2 | 0 - 123.0 | 0 - 45.8 |
| **CD8+ T Cell Subsets** | | | | | | |
| **NAÏVE** | 82.3 | 0.8 | 2.7 | 8.8 | 19.9 | 37.9 |
|  | 1.5 - 1094 | 0 - 50.8 | 0 - 1067 | 0 - 604.2 | 0 - 629.9 | 0 - 765.4 |
| **Central Memory** | 93.4 | 1.4 | 18.9 | 48.3 | 153.6 | 136.4 |
|  | 3.8 - 8166 | 0 - 238.5 | 0 - 1245 | 0 - 3052 | 0 - 2051 | 2.8 - 4376 |
| **Effector memory** | 31.5 | 2.1 | 4.9 | 7.2 | 76 | 80.5 |
|  | 0.6 - 2634 | 0 - 139.9 | 0 - 585.6 | 0 - 1053 | 0 - 2686 | 0.3 - 4466 |
| **Terminal Effector** | 73.5 | 1.04 | 1.6 | 3.5 | 27.8 | 39.2 |
|  | 0.6 - 2844 | 0 - 74.2 | 0 - 113.9 | 0 - 173.7 | 0 - 1417 | 0.1 - 1412 |

*Detailed description of immune cell counts for different immune subsets at their respective timepoints.*

Supplementary Table S2:

| **Immune cell subsets** | **No GvHD** | | | | | |
| --- | --- | --- | --- | --- | --- | --- |
|  | **PRE** | **D+14** | **D+21** | **D+28** | **D+60** | **D+90** |
| **Lymphocytes** | 1397 | 39.25 | 108.2 | 193.7 | 1049 | 1103 |
|  | 93.25 - 9085 | 1.05 - 1298 | 4.74 - 2256 | 6.5 - 4752 | 35.56 - 8504 | 8.908 - 1.619e+04 |
| **CD3+ T Cells** | 979.4 | 24.98 | 68.65 | 129.9 | 578.7 | 815.2 |
|  | 63.5 - 8604 | 0.98 - 840.6 | 3.555 - 2246 | 6 - 4699 | 14.58 - 6220 | 5.109 - 1.248e+04 |
| **B Cells** | 23.06 | 0.026 | 0 | 0.25 | 63.72 | 108.1 |
|  | 0 - 631.2 | 0 - 81.64 | 0 - 6.576 | 0 - 8.48 | 0.196 - 1359 | 0.148 - 2013 |
| **NK Cells** | 133.1 | 0.945 | 7.369 | 19.32 | 140.5 | 129.8 |
|  | 2.88 - 496.8 | 0 - 456.9 | 0.142 - 852.7 | 0.235 - 750.2 | 9.652 - 1526 | 3.406 - 1697 |
| **CD3+CD4+ T cells (Th cells)** | 427.7 | 12.48 | 18.83 | 25.85 | 104.3 | 101.7 |
|  | 9.272 - 3908 | 0.12 - 151.7 | 2.37 - 546.1 | 0.0004 - 355.6 | 1.27 - 1276 | 1.048 - 812.3 |
| **CD3+CD8+ T cells (Tc cells)** | 432.6 | 9.615 | 40.21 | 80.16 | 438.9 | 511.2 |
|  | 28.8 - 4130 | 0.28 - 507.7 | 0 - 1930 | 0.8659 - 4340 | 0 - 4549 | 4.061 - 9631 |
| **CD4+ T Cell Subsets** | | | | | | |
| **NAIVE** | 59.02 | 1.277 | 0.569 | 0.52 | 3.85 | 4.16 |
|  | 0.152 - 2427 | 0 - 75.27 | 0 - 200.5 | 0 - 323.4 | 0 - 162 | 0 - 258.1 |
| **Central Memory** | 122.4 | 4.883 | 8.604 | 14.93 | 57.38 | 64.83 |
|  | 3.8 - 932.9 | 0.036 - 67.14 | 0.79 - 239.6 | 0 - 236.6 | 0.762 - 375.8 | 0 - 527.9 |
| **Effector memory** | 43.46 | 4.123 | 5.56 | 5.72 | 33 | 34.21 |
|  | 0.48 - 503.6 | 0.06 - 71.46 | 0.049 - 116.7 | 0 - 229.5 | 0 - 1081 | 0 - 379.4 |
| **Terminal Effector** | 2.661 | 0 | 0 | 0 | 1.18 | 1.38 |
|  | 0 - 233.2 | 0 - 37.83 | 0 - 31.92 | 0 - 86.24 | 0 - 123 | 0 - 20.4 |
| **CD8+ T Cell Subsets** | | | | | | |
| **NAIVE** | 66.59 | 0.698 | 2.264 | 7.8 | 20.65 | 24.67 |
|  | 1.52 - 870.5 | 0 - 50.88 | 0 - 54.67 | 0 - 482.8 | 0 - 629.9 | 0 - 336.3 |
| **Central Memory** | 93.34 | 1.169 | 19.32 | 61.44 | 185.4 | 152.3 |
|  | 3.84 - 325 | 0 - 238.5 | 0 - 1245 | 0 - 3052 | 0 - 2051 | 2.751 - 4376 |
| **Effector memory** | 34.16 | 1.987 | 7.228 | 9.42 | 147.5 | 146.8 |
|  | 0.635 - 400.9 | 0 - 139.9 | 0 - 585.6 | 0 - 1053 | 0 - 2686 | 0.524 - 4466 |
| **Terminal Effector** | 51.61 | 1.16 | 1.917 | 4.16 | 36.67 | 38.23 |
|  | 0.635 - 2844 | 0 - 74.2 | 0 - 113.5 | 0 - 106.6 | 0 - 1417 | 0.131 - 1412 |

| **Immune cell subsets** | **2-4 aGvHD** | | | | | |
| --- | --- | --- | --- | --- | --- | --- |
|  | **PRE** | **D+14** | **D+21** | **D+28** | **D+60** | **D+90** |
| **Lymphocytes** | 1126 | 37.87 | 58.25 | 150 | 762 | 852.7 |
|  | 95.8 - 1.578e+04 | 5.475 - 403.8 | 4.982 - 1545 | 10.58 - 5773 | 5.502 - 5967 | 41.03 - 2509 |
| **CD3+ T Cells** | 883.6 | 30.21 | 52.74 | 125.6 | 599.9 | 420.4 |
|  | 77.32 - 1.493e+04 | 4.527 - 401.7 | 2.862 - 922.7 | 7.503 - 4440 | 2.49 - 2731 | 34.45 - 2371 |
| **B Cells** | 83.84 | 0.0255 | 0 | 0 | 4.05 | 45.88 |
|  | 0.42 - 1200 | 0 - 1.518 | 0 - 174.3 | 0 - 48.13 | 0 - 2831 | 0 - 686.1 |
| **NK Cells** | 120.1 | 1.525 | 7.865 | 16.19 | 153.7 | 139.9 |
|  | 18.38 - 815 | 0.2 - 33.42 | 0 - 998.6 | 0.87 - 1563 | 1.572 - 3182 | 3.679 - 656.6 |
| **CD3+CD4+ Tcells (Th cells)** | 397.6 | 20.06 | 14.95 | 32.71 | 50.94 | 50.36 |
|  | 48.64 - 2264 | 1.509 - 319.3 | 2.173 - 489.7 | 0 - 1094 | 0.249 - 1427 | 4.08 - 341.6 |
| **CD3+CD8+ T cells (Tc cells)** | 353.6 | 14.08 | 38.91 | 66.12 | 280.4 | 264.9 |
|  | 22.24 - 1.228e+04 | 1.325 - 225 | 0.53 - 371.8 | 0.1329 - 3247 | 1.572 - 2052 | 14.43 - 2227 |
|  | **CD4+ T Cell Subsets** | | | | | |
| **NAÏVE** | 150.4 | 1.854 | 1.337 | 2.316 | 2.027 | 2.435 |
|  | 7.296 - 870.1 | 0 - 42.28 | 0 - 77.72 | 0 - 137.2 | 0 - 88.49 | 0.26 - 152.3 |
| **Central Memory** | 134.1 | 11.73 | 11.71 | 21.93 | 44.26 | 29.93 |
|  | 17.42 - 1517 | 1.006 - 184.9 | 0.583 - 387.7 | 0 - 666.5 | 0 - 1177 | 2.86 - 180.2 |
| **Effector memory** | 43.05 | 4.981 | 3.404 | 3.932 | 6.93 | 16.06 |
|  | 1.82 - 504.8 | 0.503 - 90.72 | 0 - 78.39 | 0 - 251.6 | 0 - 241.2 | 0.68 - 188.4 |
| **Terminal Effector** | 2.73 | 0.1025 | 0 | 0 | 0.168 | 0.65 |
|  | 0.14 - 21.11 | 0 - 3.036 | 0 - 9.38 | 0 - 29.4 | 0 - 11.3 | 0 - 45.76 |
|  | **CD8+ T Cell Subsets** | | | | | |
| **NAÏVE** | 134.6 | 1.165 | 3.054 | 13.53 | 15.54 | 72.18 |
|  | 7.54 - 1094 | 0 - 15.94 | 0 - 49.88 | 0.122 - 604.2 | 0 - 211.9 | 0.53 - 765.4 |
| **Central Memory** | 93.93 | 4.921 | 18.95 | 38.39 | 126 | 100.2 |
|  | 12.64 - 8186 | 0.26 - 71.3 | 0.106 - 328.7 | 2.318 - 2052 | 0.249 - 1016 | 3.679 - 984 |
| **Effector memory** | 22.68 | 1.68 | 4.075 | 4.275 | 40.5 | 41.32 |
|  | 1.14 - 2634 | 0.234 - 112.5 | 0 - 75.71 | 0 - 937.6 | 0.249 - 534.6 | 0.283 - 245.2 |
| **Terminal Effector** | 102.8 | 0.8375 | 0.909 | 2.858 | 15.54 | 40.08 |
|  | 0.88 - 383.5 | 0 - 52.37 | 0 - 113.9 | 0 - 173.7 | 0 - 1309 | 0.283 - 428.8 |

*Comparison of IR pattern between patients with and without grade 2-4 aGvHD*

Supplementary Table S3 : Immune subsets associated with the occurrence of Grade2-4 acute GVHD

| **Time points** | **T cell subsets** | **No GvHD (Median)** | **2-4 aGvHD**  **(Median)** | **p value** |
| --- | --- | --- | --- | --- |
| **Neutrophil engraftment (ANC)** | CM CD8+ Tcells | 1.169 | 4.921 | 0.066 |
|  |  | (0 - 238.5) | (0.26 - 71.2) |  |
| **at Day +28** | CM CD4+ T cells | 14.93 | 21.93 | 0.057 |
|  |  | (0 - 236.6) | (0 - 666.5) |  |

*Absolute counts are measured in cells/µl with median and range. 2-4 aGvHD includes all patients with Overall Grade 2-4 GVHD. No GVHD group includes patients with No GVHD and Grade 1 GVHD. EM – Effector memory; CM – Central memory; TD – Terminal effector.*

Supplementary Table S4: Immune subsets associated with the occurrence of Grade3-4 acute GVHD

| **Time points** | **T cell subsets** | **No GvHD (Median)** | **3-4 aGvHD**  **(Median)** | **p value** |
| --- | --- | --- | --- | --- |
| **at Day +21** | Naïve CD4+ T cells | 0.62 | 1.67 | 0.055 |
|  |  | (0 - 200.4) | (0 - 25.3) |  |
|  | CM CD4+ T cells | 7.48 | 19.7 | 0.052 |
|  |  | (0.58 - 239.6) | (1.71 - 387.6) |  |
| **at Day +28** | TD CD4+T cells | 2.3 | 0.04 | 0.019 |
|  |  | (0 - 86.2) | (0 - 0.61) |  |
|  | EM CD8+T cells | 8.6 | 3.15 | 0.021 |
|  |  | (0 - 1053.1) | (0 -97.2) |  |

*Absolute counts are measured in cells/µl with median and range. 3-4 aGvHD includes all patients with Overall Grade3-4 GVHD. No GvHD group includes patients with No GVHD, Grade 1 GVHD and Grade 2 GVHD. EM – Effector memory; CM – Central memory; TD – Terminally effector.*

Supplementary Table S5: Reconstitution kinetics of T-cell and regulatory T-cell subsets.

| **Immune subset** | **Donor** | **ANC** | **Day+28** | **Day+60** | **Day+90** |
| --- | --- | --- | --- | --- | --- |
| **CD3+ T Cells** | 2071 | 52.94 | 164.6 | 1007 | 983.4 |
|  | 657.3 - 14175 | 0 - 790.9 | 0 - 8608 | 60.73 - 5512 | 86.77 - 5899 |
| **CD3+CD4+ Tcells (Th cells)** | 812 | 22.46 | 24.26 | 150.4 | 250.4 |
|  | 194.9 - 6518 | 2.158 - 166.8 | 0.3328 - 3826 | 3.243 - 1279 | 11.94 - 1663 |
| **CD4+CD25+CD127- Tregs** | 33.77 | 3.638 | 3.053 | 8.853 | 10.91 |
|  | 0 - 220 | 0.2661 - 41.61 | 0.05556 - 920.7 | 0.8778 - 179.8 | 1.731 - 72.76 |
| **CD4+CD25+CD127- FOXP3+Tregs** | 31.95 | 2.966 | 3.23 | 8.286 | 10.42 |
|  | 4.67 - 208.1 | 0.1141 - 41.82 | 0.05755 - 57.83 | 0 - 73.27 | 0 - 75.23 |

*Absolute counts of T-cell and regulatory T-cell (Treg) subsets are presented as median cells/µL, with ranges shown below each value. Donor values represent immune subset counts in the corresponding stem cell graft samples and are provided as a reference for post-transplant recovery. ANC = absolute neutrophil count*

Supplementary Table S6: Comparison of immune reconstitution pattern of Tregs between patients with and without aGvHD.

| **Immune subset** | **No GvHD** | | | | |
| --- | --- | --- | --- | --- | --- |
|  | **Donor** | **ANC** | **Day+28** | **Day+60** | **Day+90** |
| **CD3+ T Cells** | 2083.4895 | 38.68 | 151 | 923.1 | 1542 |
|  | 657.25 - 14175.38 | 3.37 - 790.9 | 3.37 - 2407 | 60.74 - 4643 | 86.77 - 5899 |
| **CD3+CD4+ Tcells (Th cells)** | 858.159 | 13.18 | 24.26 | 146.5 | 250.4 |
|  | 194.8 - 6517.9 | 0.3328 - 162 | 0.3328 - 948.1 | 3.243 - 1279 | 0 - 3186 |
| **CD4+CD25+CD127- Tregs** | 37.84032189 | 2.766 | 4.266 | 8.516 | 8.772 |
|  | 0 - 220.04 | 0.1216 - 41.61 | 0.05556 - 47.4 | 0.8778 - 97.42 | 1.731 - 38.69 |
| **CD4+CD25+CD127- FOXP3+Tregs** | 33.26817312 | 2.475 | 4.855 | 8.748 | 9.935 |
|  | 4.6 - 208.57 | 0.1141 - 41.82 | 0.05755 - 57.83 | 0.9154 - 73.27 | 1.723 - 75.23 |

| **Immune subset** | **Yes GvHD** | | | | |
| --- | --- | --- | --- | --- | --- |
|  | **Donor** | **ANC** | **Day+28** | **Day+60** | **Day+90** |
| **CD3+ T Cells** | 1732.1525 | 58.95 | 188.4 | 1460 | 2029 |
|  | 848.9 - 3974.1 | 6.577 - 469.9 | 20.75 - 1126 | 82.29 - 5512 | 206.9 - 5406 |
| **CD3+CD4+ Tcells (Th cells)** | 724.68 | 23.49 | 47.23 | 169.8 | 355 |
|  | 261.8 - 1675.7708 | 3.168 - 166.8 | 4.058 - 310.7 | 32.46 - 1170 | 42.77 - 1663 |
| **CD4+CD25+CD127- Tregs** | 32.6106 | 3.451 | 2.713 | 7.8 | 19.97 |
|  | 9.58056 - 103.76 | 0.2661 - 16.39 | 0.0814 - 58.6 | 3.457 - 55 | 4.074 - 48.15 |
| **CD4+CD25+CD127- FOXP3+Tregs** | 28.9872 | 3.128 | 3.091 | 7.819 | 14.69 |
|  | 7.57344 - 115.3 | 0.2281 - 21.67 | 0.1628 - 47.63 | 3.808 - 53.34 | 2.841 - 45.04 |

*Absolute counts of T-cell and regulatory T-cell (Treg) subsets are presented as median cells/µL, with ranges shown below each value. ANC; Absolute neutrophil count.*

**Supplementary Figure S1**

*Figure S1. Absolute lymphocyte count recovery according to conditioning intensity (MAC vs RIC). Median ALC values are shown at pre-transplant, ANC recovery, and days +21, +28, +60, and +90 post-transplant. Error bars represent interquartile range. A) Comparison of ALC recovery kinetics between patients receiving myeloablative conditioning (MAC) and reduced-intensity conditioning (RIC) in the overall cohort (n = 95). (B) Comparison of T cell recovery kinetics between MAC and RIC recipients in the Treg sub-analysis (n = 50).*

**Supplementary Figure S2**

***Figure S2.*** *Absolute standardized mean differences (SMD) for baseline covariates after propensity-score matching (63 pairs). Blue points indicate SMD <0.1 and red points SMD ≥0.1; the dashed line marks the prespecified threshold of 0.1. After matching, age, sex, marrow failure and lymphoid malignancy (absent in both arms) were balanced, whereas "Other" diagnoses (SMD 0.68), myeloid malignancy (0.54), CD34 dose (0.20) and HCT-CI (0.12) exceeded the threshold.*
